# Prolonged Continuous Theta Burst Stimulation Can Regulate Sensitivity on Aβ Fibers: An Functional Near-Infrared Spectroscopy Study

**DOI:** 10.3389/fnmol.2022.887426

**Published:** 2022-04-12

**Authors:** Chong Li, Nannan Zhang, Qiong Han, Lifang Zhang, Shuo Xu, Shuting Tu, Yong Xie, Zhiyong Wang

**Affiliations:** Department of Rehabilitation Medicine, First Affiliated Hospital of Fujian Medical University, Fujian, China

**Keywords:** repetitive transcranial magnetic stimulation, theta-burst stimulation, pain, sensory fiber, functional near-infrared spectroscopy

## Abstract

**Objective:**

High-frequency repetitive transcranial magnetic stimulation (rTMS) induces analgesic effects in both experimental pain and clinical pain conditions. However, whether rTMS can modulate sensory and pain thresholds on sensory fibers is still unclear. Here, we compared the effects of three rTMS paradigms on sensory and pain thresholds conducted by different sensory fibers (Aβ, Aδ, and C fibers) with sham stimulation and investigate the potential brain activation using functional near-infrared spectroscopy (fNIRS).

**Methods:**

Forty right-handed healthy subjects were randomly allocated into one of four groups. Each subject received one session rTMS [prolonged continuous theta-burst stimulation (pcTBS), intermittent theta-burst stimulation (iTBS), 10 Hz rTMS or sham]. Current perception threshold (CPT), pain tolerance threshold (PTT), and fNIRS were measured at baseline, immediately after stimulation, and 1 h after stimulation, respectively.

**Results:**

Significant differences between treatments were observed for changes for CPT 2,000 Hz between baseline and 1 h after rTMS (*F* = 6.551, *P* < 0.001): pcTBS versus sham (*P* = 0.004) and pcTBS versus 10 Hz rTMS (*P* = 0.007). There were significant difference in average HbO μm in the right frontopolar cortex (FPC) [channel 23: *P* = 0.030 (pcTBS versus sham: *P* = 0.036)], left dorsolateral prefrontal cortex (DLPFC) [channel 7: *P* = 0.006 (pcTBS versus sham: *P* = 0.004)], left FPC [channel 17: *P* = 0.014 (pcTBS versus sham: *P* = 0.046), channel 22: *P* = 0.004 (pcTBS versus sham: *P* = 0.004)] comparing four group in 1 h after stimulation in PTT 2000 Hz (Aβ-fiber).

**Conclusion:**

Prolonged continuous theta-burst stimulation can regulate sensitivity on Aβ fibers. In addition, single-session pcTBS placed on left M1 can increase the excitability of DLPFC and FPC, indicating the interaction between M1 and prefrontal cortex may be a potential mechanism of analgesic effect of rTMS. Studies in patients with central post-stroke pain are required to confirm the potential clinical applications of pcTBS.

## Introduction

For the revised International Association for the Study of Pain definition, pain is termed as an unpleasant sensory and emotional experience associated with, or resembling that associated with, actual or potential tissue damage (Raja et al., 2020). Pain is mainly transmitted by sensory nerve fibers. According to morphological, electrophysiological, and functional characteristics, sensory nerve fibers can be divided into three main subgroups: large myelinated sensory nerve fibers (Aβ fibers), small myelinated sensory nerve fibers (Aδ fibers), and unmyelinated sensory nerve fibers (C fibers) (Lynn and Carpenter, 1982). In the peripheral nerves, vibration sensation, tactile sensation, and light pressure sensation are mainly conducted by Aβ fibers. Temperature sensation, rapid pain sensation, and pressure sensation are mainly conducted by Aδ fibers. Warmth, slow pain, and various forms of nociceptive sensation are mainly conducted by C fibers (Schmelz, 2011; Paricio-Montesinos et al., 2020; Gomatos and Rehman, 2022). Pain is a subjective emotional experience that may be due to many different diseases or conditions, and there are few effective treatments. At present, the application of analgesic drugs is the main way to relieve pain (Finnerup et al., 2015, 2021). However, long-term use of analgesic drugs is not only prone to addiction, but also has many side effects (Koob, 2021).

Transcranial magnetic stimulation (TMS) is a biological stimulation technology that uses the time-varying magnetic field to act on the cerebral cortex to generate induced current and change the action potential of cortical nerve cells, thus affecting brain metabolism and nerve electrical activity (Cárdenas-Morales et al., 2010; Lefaucheur, 2019). Repeated transcranial magnetic stimulation (rTMS) refers to the process of giving repeated stimulation to a specific cortical area. As a painless, safe, and non-invasive brain stimulation technology, rTMS is gradually applied to pain therapy caused by various conditions (Lefaucheur et al., 2014, 2020; Klein et al., 2015). It has been shown that high frequency (>5 Hz) rTMS applied over the primary motor cortex (M1) can induce analgesic effects against both experimental pain (Summers et al., 2004; Nahmias et al., 2009; Houzé et al., 2013) and chronic pain (André-Obadia et al., 2008; Young et al., 2014; Attia et al., 2021). Studies indicated that the analgesic effect of 10 Hz rTMS is better than that of other frequencies (André-Obadia et al., 2008; Young et al., 2014; Attia et al., 2021). In addition, studies have shown that rTMS may relieve pain by regulating neural plasticity, influencing cerebral blood flow changes, and mediating pain circuits (Hoogendam et al., 2010; Dall’Agnol et al., 2014; Park et al., 2017).

In addition to the classic rTMS, new rTMS parameters have been described. Theta burst stimulation (TBS) consists of bursts of three pulses at 50 Hz, repeated five times per second. Intermittent TBS (iTBS) with 600 pulses and prolonged continuous TBS (pcTBS) with 1,200 pulses induce facilitation of cortical excitability (Huang et al., 2005; Gamboa et al., 2010; Moisset et al., 2015). Such stimulation sessions are much shorter than classical high-frequency rTMS sessions, which can optimize medical resources. Existing studies mainly focused on the effects of different parameters of rTMS on different experimental pain (such as cold pain, hot pain, tenderness, etc.) and clinically related pain, such as post-stroke pain. However, the analgesic effect of rTMS with different parameters on sensory fibers remains unclear.

Therefore, we hypothesized that pcTBS and/or iTBS would yield analgesic effects and modulate sensitivity on sensory fibers similar to or, stronger than classical 10 Hz rTMS. We carried out a double-blind, randomized controlled study in healthy volunteers to prove our hypothesis. The purpose of this study is twofold: first, to compare the effects of the three rTMS paradigms on pain thresholds conducted by different sensory fibers; and second, to investigate the potential mechanisms of action of these stimulation paradigms using functional near-infrared spectroscopy (fNIRS).

## Materials and Methods

### Study Design

This was a double-blind, four-group randomized controlled trial comparing three types of rTMS with sham stimulation on sensory and pain threshold in healthy volunteers. The protocol involved four experimental sessions, in which we compared the effects of pcTBS, iTBS, 10 Hz rTMS, and sham stimulation on sensory and pain thresholds. In each session, the stimulation administered targeted the left primary motor cortex. This study was conducted at the first affiliated hospital of Fujian Medical University (Fujian, China) from November 2021 to January 2022 and was approved by the Institutional Review Board of Huashan Hospital, Fudan University (KY2021-815).

### Participants

Forty healthy volunteers were recruited in this study. The inclusion criteria were: (1) right-handed non-smokers; (2) aged between 20 and 40; and (3) free of pain during the past 6 months. The exclusion criteria included: (1) a history of chronic pain or recent acute pain; (2) on medication at the time of testing or during the previous; (3) serious medical conditions; (4) pregnancy or breastfeeding; (5) sensory impairment. All of the participants gave written informed consent after inclusion.

### Experimental Procedures

A blinded evaluator performed assessments for all participants. All participants were assessed sensory and pain thresholds after inclusion. An independent researcher not involved in the study created a blocked randomization sequence using a computerized program (Microsoft Excel). Block randomization ensured equal numbers of participants for group allocation. Allocation assignments were placed in sequentially numbered, opaque, and sealed envelopes by an offsite officer not involved in the study. Participants were blind regarding the intervention received. Once the participant completed the baseline assessment, an independent person would open an envelope and reveal the group allocation.

**FIGURE 1:**
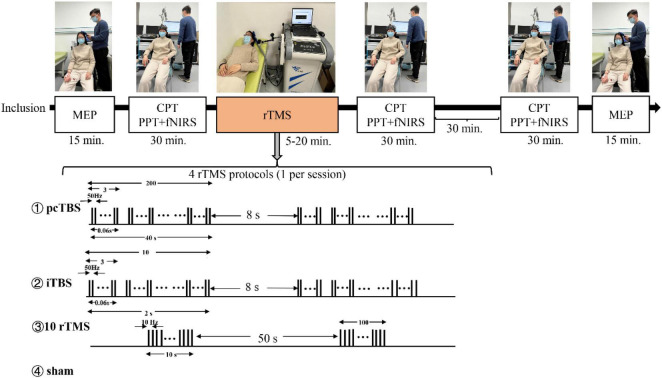
Schematic overview of the whole study. MEP means motor evoked potentials, CPT means current perception thresholds, PTT means pain tolerance threshold, fNIRS means functional near-infrared spectroscopy. Prolonged continuous theta-burst stimulation (pcTBS) consisted of three pulses at 50 Hz repeated 400 times at intervals of 200 ms (1,200 pulses, 1 min and 40 s). Intermittent theta-burst stimulation (iTBS) consisted of three pulses at 50 Hz repeated 10 times at intervals of 200 ms (600 pulses, 3 min and 20 s). 10 Hz rTMS consisted of 15 trains of 10 s with an interval of 50 s (1,500 pulses, 15 min).

After giving informed consent, participants were allocated to one of four groups receiving one session of rTMS stimulation. The evaluator would assess sensory and pain thresholds for all participants immediately after stimulation and 1 h after stimulation (Figure 1).

### Transcranial Magnetic Stimulation

A magnetic therapy device (Model CCY-II; Wuhan Yiruide Medical Equipment Co., Ltd., Wuhan, China, YZB-20142211249) was used. rTMS was applied over the left M1 using a figure-of-eight-shaped coil (70 mm diameter) positioned tangentially to the scalp and horizontally in the posterior-anterior direction, which proved to be effective in pain relief (Andre-Obadia et al., 2018).

Resting motor threshold (RMT) was determined experimentally as the lowest stimulation intensity that produced motor evoked potentials (MEP) ≥ 50 μV in 50% of trials (Rossini et al., 1994). In addition, we also recorded the cortical latency of the subjects.

Repetitive transcranial magnetic stimulation was applied at 80% of the RMT, as in previous studies in which that was sufficient to induce pain analgesia in healthy volunteers (Nahmias et al., 2009; de Andrade et al., 2011). Three active and one sham stimulation were applied randomly, with only one type of stimulation applied for each participant. pcTBS consisted of three pulses at 50 Hz repeated 400 times at intervals of 200 ms (1,200 pulses, 1 min and 40 s). iTBS consisted of three pulses at 50 Hz repeated 10 times at intervals of 200 ms (600 pulses, 3 min and 20 s). The 10 Hz rTMS pattern consisted of 15 trains of 10 s with an interval of 50 s (1,500 pulses, 15 min).

### Outcome Measures

#### Sensory and Pain Thresholds Assessments

**FIGURE 2:**
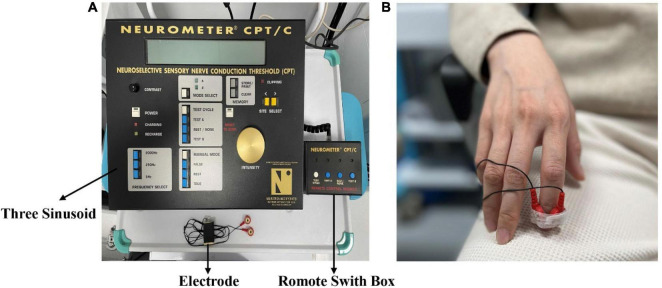
**(A)** The Neurometer^®^ device. **(B)** The electrode is placed on the fingertip of the right index finger.

Painless current perception threshold (CPT) and pain tolerance threshold (PTT) were evaluated using Neurometer^®^ device (the UAS). The Neurometer^®^ generates a constant current stimulus which evokes responses that quantify the functional integrity of each of the three major sub-populations of sensory nerve fibers. Specifically, Aβ, Aδ, and C fiber groups are selectively stimulated by sinusoid waveform currents of 2,000, 250, and 5 Hz respectively. Using small surface electrodes, this test generated discrete double-blinded CPT measures (*P* < 0.006). After the start of the test, the subjects were placed in a comfortable position and a pair of electrodes were fixed to the tip of the index finger of the subject’s right hand with adhesive tape (Figure 2).

For the CPT test, the Neurometer^®^ device emits stimuli of three different frequencies. Subjects need to distinguish between “true” and “false” stimuli randomly generated by the detector. After a sufficient number of consistent tests are conducted for each stimulus frequency, the detector determines the current sensing threshold of the frequency test.

When the intensity of Neurometer^®^ stimulation exceeds the painless CPT value, it will induce pain. Under the self-control of the subjects, the maximum intensity that can tolerate nerve selective electrical stimulation is defined as the pain tolerance threshold. The PTT was tested combined with the functional near-infrared test.

#### Functional Near-Infrared Spectroscopy Neuroimaging and Probe Localization

**FIGURE 3:**
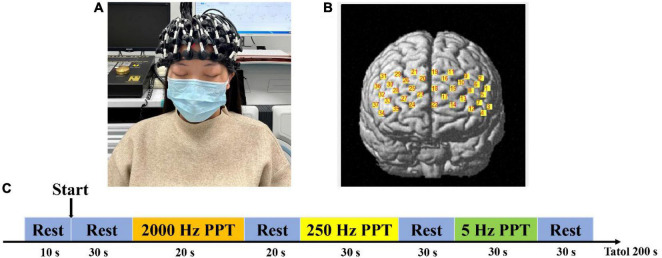
Illustration of fNIRS testing. **(A)** Wearing method of functional near-infrared spectroscopy cap. **(B)** Brain localization schema of channels. **(C)** fNIRS testing procedure. PTT means pain tolerance threshold.

We used an fNIRS system (BS-3000, Wuhan Znion Technology Co., Ltd., Wuhan, China) with wavelengths of 695 and 830 nm. The fNIRS cap setup included 12 emitters of near-infrared light and 12 detectors spaced 3 cm apart, yielding 37 data channels deployed at the prefrontal area according to the EEG-10-20 system (Figures 3A,B). The system used a chin strap to secure the cap in place to reduce cap movement. A NIR gain quality check was performed to ensure data acquisition before recording. Neuroimaging data were collected at a sampling rating of 20 Hz throughout the entire experiment.

For each fNIRS testing, participants were asked to rest for 30 s, followed by the PTT tests (20 s 2,000 Hz PTT test and 20 s rest, 30 s 250 Hz PTT test and 30 s rest, and 30 s 5 Hz PTT test and 30 s rest) (Figure 3C). Using fNIRS, we observed the activation of different parameters of rTMS applied on the left M1 in the prefrontal cortex (Broca’s area, dorsolateral prefrontal cortex, frontopolar area, and orbitofrontal area).

### Statistical Analysis

Statistical analysis was performed in IBM SPSS Statistics (version 26). Data were confirmed to have a normal distribution using the Shapiro–Wilk normality test since the sample size was small. A repeated-measures analysis of variance (ANOVA) with the factors “time,” “stimulation,” and the “time × stimulation” interaction was used for the comparison of CPT, PTT. If there was a significant “time × stimulation,” simple main effects were calculated for “time” and “stimulation” and paired *t*-tests with Bonferroni’s correction for multiple comparisons. One-way ANOVA was used for comparison between four groups. For categorical variables, we use Fisher exact test to compare differences between groups.

In order to minimize motion artifact and ambient light noise for fNIRS data, a low pass filter was used to filtered the detected signals. Modified Beer-Lambert Law (MBLL) was used to calculate hemodynamic changes for each of the 37 channels. To demonstrate the differences in the prefrontal cortex activity, NIRS_SPM and Homer_2 were used the make topographical maps. A repeated-measures ANOVA was used to calculate difference for intra-group comparison. One-way ANOVA was used to calculate difference for inter-group comparison. Statistical significance was established at *P* < 0.05.

## Results

Forty healthy volunteers were included in the study (mean age: 24.7 ± 4.43 years, 26 men and 14 women) to receive different rTMS parameters applied to the left M1. None volunteer withdrew from the study. The participants’ baseline demographic characteristics are shown in Table 1. No significant differences were observed between the groups regarding gender, age, RMT, MEP-latency, CPT, and PTT (*P* > 0.05).

**TABLE 1:** Baseline information for four groups.

Variable	pcTBS	iTBS	10 Hz rTMS	sham	F/*X*^2^	*P*-value
Age (years)	26.9 ± 6.45	24.7 ± 4.19	24.5 ± 3.53	22.7 ± 1.88	1.573	0.213
Gender, *n*					0.424	0.737
Man/Female	3/7	4/6	4/6	3/7		
BMI (kg/m^2^)	20.97 ± 3.52	21.94 ± 3.75	21.95 ± 3.36	22.69 ± 3	0.441	0.932
RMT (percentage)	38 ± 13.3	39 ± 7.3	36 ± 10.4	34 ± 7	0.452	0.717
MEP-latency (ms)	24.34 ± 1.71	25.02 ± 2.01	24.95 ± 3.62	23.25 ± 1.94	1.120	0.354
CPT-2 kHz	147.40 ± 36.13	144.40 ± 30.32	134.60 ± 27.62	155.30 ± 22.44	0.839	0.482
CPT-250 Hz	65.60 ± 24.41	51 ± 17.49	55 ± 13.36	64.9 ± 20.81	1.395	0.260
CPT-5 Hz	37.4 ± 17.31	29.9 ± 13.15	28.8 ± 11.81	37.5 ± 17.23	0.971	0.417
PTT-2 kHz	11.3 ± 4.11	10.3 ± 4.54	10.5 ± 3.81	11.1 ± 5.15	0.115	0.951
PTT-250 Hz	7.3 ± 2.79	7.8 ± 4.8	6.9 ± 2.99	7.23 ± 3.23	0.163	0.921
PTT-5 Hz	12.7 ± 5.31	10.3 ± 5.22	10.7 ± 5.18	11.5 ± 5.64	0.392	0.760

*Data are presented as mean ± SD. pcTBS, prolonged continuous theta-burst stimulation; iTBS, intermittent theta-burst stimulation; rTMS, repetitive transcranial magnetic stimulation; BMI, body mass index; RMT, resting motor thresholds; MEP, motor evoked potential; CPT, current perception threshold; PTT, pain tolerance threshold.*

### Effect of Left M1 Repetitive Transcranial Magnetic Stimulation on Sensory Threshold

**FIGURE 4:**
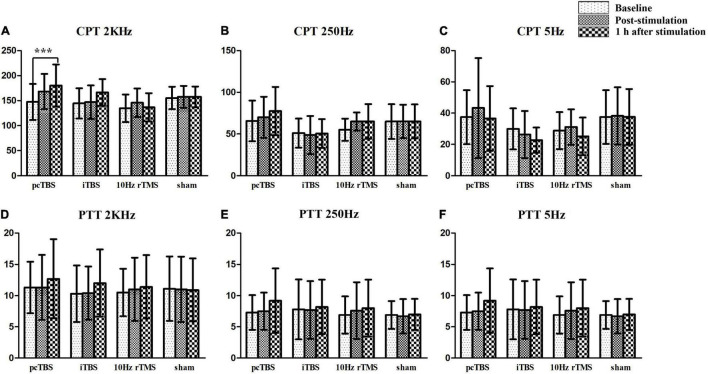
Sensory and pain thresholds measurements. CPT means current perception threshold and PTT means pain tolerance threshold. pcTBS, prolonged continuous theta-burst stimulation; iTBS, intermittent theta-burst stimulation; rTMS, repetitive transcranial magnetic stimulation. ***P < 0.001.

**FIGURE 5:**
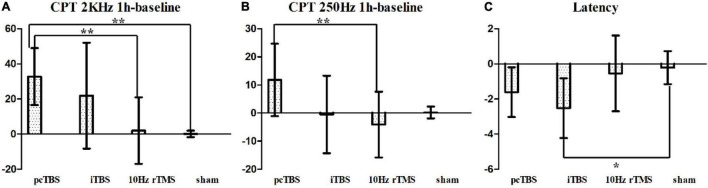
**(A)** Changes in current perception threshold of 2,000 Hz between baseline and 1 h after stimulation. **(B)** Changes in current perception threshold of 250 Hz between baseline and 1 h after stimulation. **(C)** Changes of latency in MEP between baseline and 1 h after stimulation. pcTBS, prolonged continuous theta-burst stimulation; iTBS, intermittent theta-burst stimulation; rTMS, repetitive transcranial magnetic stimulation. *P < 0.05; **P < 0.001 (not significant otherwise).

Sensory thresholds and pain thresholds were determined for the right index fingertip, which represented the global effect of stimulation. Significant differences between treatments were observed for changes in CPT 2K Hz between baseline and 1 h after rTMS (*F* = 6.551, *P* < 0.001): pcTBS versus sham (*P* = 0.004) and pcTBS versus 10 Hz rTMS (*P* = 0.007) (Figure 5A). There was a significant difference for changes in CPT 250 Hz between baseline and 1 h after stimulation (*F* = 3.809, *P* = 0.018): pcTBS versus 10 Hz rTMS (*P* = 0.018) (Figure 5B). No significant effect of treatments was observed for CPT 2K Hz (*F*(time × stimulation) = 3.127, *P* = 0.058), CPT 250 Hz (*F*(time × stimulation) = 2.286, *P* = 0.082), CPT 5 Hz (*F*(time × stimulation) = 1.312, *P* = 0.268) (Table 2 and Figures 4A–C).

**TABLE 2:** Comparison of outcomes in the four groups in post-stimulation and 1 h after stimulation.

Variable	pcTBS	iTBS	10Hz rTMS	sham	F/*X*^2^	*P*-value
RMT-1 h (percentage)	35 ± 11.6	38 ± 7.4	34 ± 10.1	34 ± 6.3	0.384	0.765
MEP-latency 1 h	22.73 ± 1.93	22.50 ± 1.27	24.41 ± 2.77	23.04 ± 1.89	1.757	0.173
CPT-2K Hz post	168.2 ± 35.24	147 ± 33.57	145.7 ± 28.56	157.5 ± 21.94	1.201	0.323
CPT-2K Hz 1h	180.2 ± 42.2	166.3 ± 26.82	136.6 ± 28.39	157.4 ± 20.7	3.577	0.023*
CPT-250 Hz post	69.9 ± 24.76	48.7 ± 22.86	52.1 ± 13.45	65 ± 20.07	2.391	0.085
CPT-250 Hz 1 h	77.4 ± 28.99	50.5 ± 17.27	50.9 ± 10.96	65.1 ± 20.38	3.964	0.015*
CPT-5 Hz post	43.4 ± 31.91	26.3 ± 15.03	31.1 ± 11.35	38.2 ± 18.39	1.335	0.278
CPT-5 Hz 1 h	36.6 ± 20.7	22.7 ± 8.09	25.1 ± 12.04	37.6 ± 17.85	2.473	0.077
PTT-2K Hz post	11.3 ± 5.2	10.4 ± 4.27	11 ± 5.05	11 ± 5.22	0.058	0.981
PTT-2K Hz 1 h	12.7 ± 6.32	12 ± 5.37	11.4 ± 5.08	10.9 ± 5.04	0.201	0.895
PTT-250 Hz post	7.5 ± 2.99	7.7 ± 4.64	7.6 ± 4.55	6.7 ± 2.75	0.142	0.934
PTT-250 Hz 1 h	9.2 ± 5.18	8.2 ± 4.36	8 ± 4.57	7 ± 2.49	0.446	0.722
PTT-5 Hz post	12.8 ± 4.87	10.7 ± 4.49	10.4 ± 4.57	10.9 ± 6.22	0.455	0.715
PTT-5 Hz 1 h	13.9 ± 6.91	10 ± 4.66	11.7 ± 5.16	11.3 ± 6.03	0.792	0.506

*Data are presented as mean ± SD. pcTBS, prolonged continuous theta-burst stimulation; iTBS, intermittent theta-burst stimulation; rTMS, repetitive transcranial magnetic stimulation; RMT, resting motor thresholds; MEP, motor evoked potential; CPT, current perception threshold; PTT, pain tolerance threshold.*

*P < 0.05.

### Effect of Left M1 Repetitive Transcranial Magnetic Stimulation on Pain Threshold

There was no significant difference on pain threshold for the test stimulation for PTT 2K Hz [*F*(stimulation) = 0.072, *P* = 0.974], PTT 250 Hz [*F*(stimulation) = 0.214, *P* = 0.886], and PTT 5Hz [*F*(stimulation) = 0.556, *P* = 0.649] (Figures 4D–F). Neither effect of change for PTT 2K Hz (*F* = 0.706, *P* = 0.554), PTT 250 Hz (*F* = 1.085, *P* = 0.368), PTT 2K Hz (*F* = 0.751, *P* = 0.529) between baseline and 1 h after stimulation.

### Effect of Left M1 Repetitive Transcranial Magnetic Stimulation on Left M1 Cortical Excitability

The mean baseline RMT was 37 ± 9.6% of the stimulator maximum output power [*F*(stimulation) = 0.452, *P* = 0.717]. There was no significant change after stimulation (*F* = 1.420, *P* = 0.253) (Table 2). The latency of the RMT was 24.39 ± 2.459 ms at baseline [*F*(stimulation) = 1.120, *P* = 0.354]. Significant difference was observed for change for latency (*F* = 4.260, *P* = 0.011): iTBS versus sham (*P* = 0.017) (Figure 5C).

### Effect of Left M1 Repetitive Transcranial Magnetic Stimulation on Brain Activation

**FIGURE 6:**
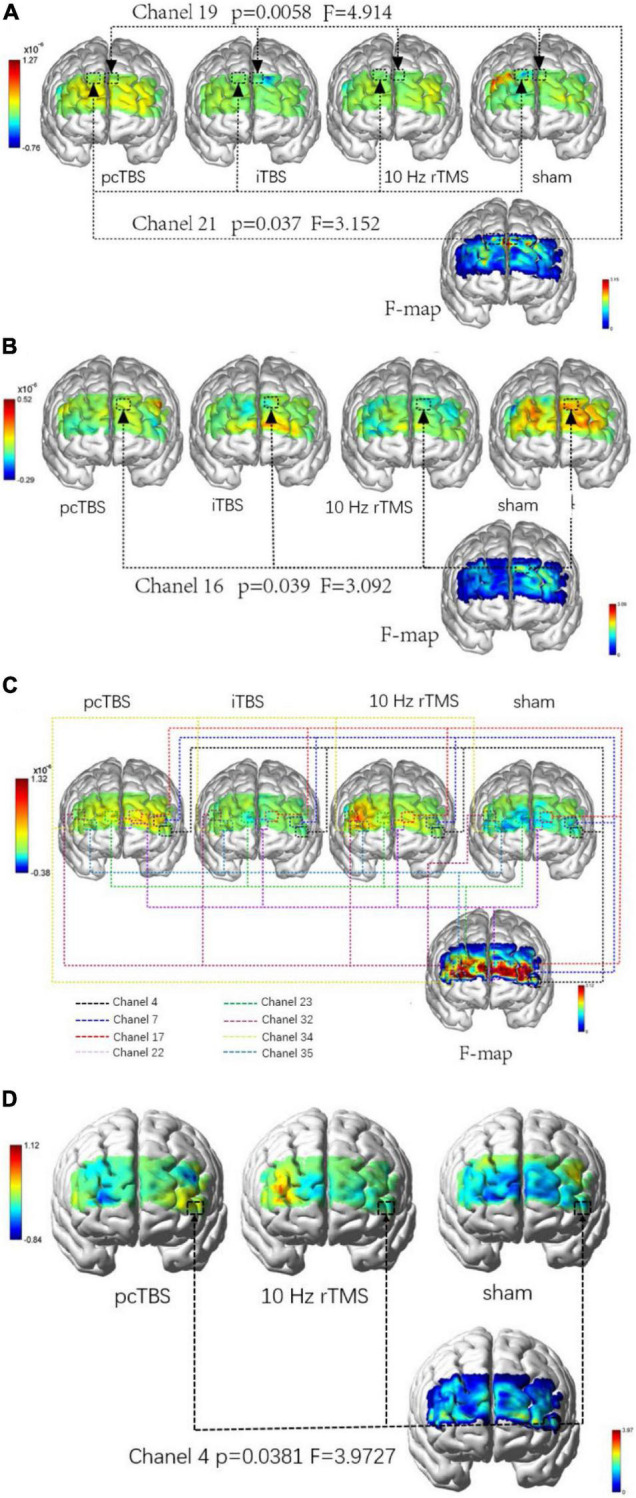
**(A)** Comparison of difference in brain activation when performing PTT 2,000 Hz task immediately after stimulation. **(B)** Comparison of difference in brain activation when performing PTT 5 Hz task immediately after stimulation. **(C)** Comparison of difference in brain activation when performing PTT 2,000 Hz task 1 h after stimulation. **(D)** Comparison of difference in changes between 1 h after stimulation and baseline in brain activation when performing PTT 2,000 Hz. pcTBS, prolonged continuous theta-burst stimulation; iTBS, intermittent theta-burst stimulation; rTMS, repetitive transcranial magnetic stimulation.

There were no significant differences in average oxygenated hemoglobin (HbO) among four groups in baseline (*P* > 0.05). There was significant difference in average HbO μm in the right dorsolateral prefrontal cortex (DLPFC) [*P* = 0.037 (pcTBS versus sham: *P* = 0.041)] and left DLPFC [*P* = 0.0058 (pcTBS versus iTBS: *P* = 0.005, pcTBS versus 10 Hz rTMS: *P* = 0.034)] when performing PTT 2,000 Hz task immediately after stimulation (Figure 6A). Significant difference was found in average HbO μm in left DLPFC [*P* = 0.039 (iTBS versus sham: *P* = 0.024)] when performing PTT 5 Hz task immediately after stimulation (Figure 6B). There was significant difference in average HbO μm in the right DLPFC [channel 32: *P* = 0.038 (10 Hz rTMS versus sham: *P* = 0.032), channel 34: *P* = 0.020 (10 Hz rTMS versus sham: *P* = 0.013)], right frontopolar cortex (FPC) [channel 23: *P* = 0.030 (pcTBS versus sham: *P* = 0.036), channel 35: *P* = 0.008 (10 Hz rTMS versus sham: *P* = 0.005)], left DLPFC [channel 7: *P* = 0.006 (pcTBS versus sham: *P* = 0.004)], left FPC [channel 17: *P* = 0.014 (pcTBS versus sham: *P* = 0.046), channel 22: *P* = 0.004 (pcTBS versus sham: *P* = 0.004)] (Figure 6C) comparing four group in 1 h after stimulation of PTT 2K Hz. When comparing the changes of PPT 2K Hz between 1 h after stimulation and baseline, significant difference was found in average HbO um in left DLPFC (F = 3.9727, *P* = 0.038): pcTBS versus sham: *P* = 0.029 (Figure 6D).

### Adverse Effects

Mild headaches occurred in one subject after pcTBS and one subject after iTBS stimulation. No serious adverse effects occurred.

## Discussion

This study aimed to identify the sensory thresholds, pain thresholds, and functional brain activity changes by comparing three rTMS paradigms with sham stimulation. The results of this study indicate that pcTBS can modulate sensitivity on Aβ fibers compared with 10 Hz rTMS and sham stimulation. In addition, pcTBS applied to left M1 can activate DLPFC and FPC after stimulation compared with iTBS, 10 Hz rTMS, and sham stimulation. However, no significant changes were found in pain tolerance threshold from behavioral data.

### Acute Sensory and Pain Threshold Change After rTMS Stimulation

Our results revealed that one-session pcTBS can modulate sensitivity on Aβ fibers compared with 10 Hz rTMS and sham stimulation. In addition, on-session pcTBS can modulate sensitivity on Aδ fibers compared with iTBS. This result is congruent with the statement that doubling the stimulation duration of the cTBS can convert inhibitory cTBS into facilitatory pcTBS (Gamboa et al., 2010; De Martino et al., 2019). In addtion, one study indicated that 1 Hz rTMS over M1 had significant modulatory effects on pain perception (Tamura et al., 2004a). Our study found that pcTBS (50 Hz) can increase the Aβ-fiber threshold, which can support the previous studies. pcTBS has some potential advantages over other parameters. On the one hand, pcTBS can promote cortical excitability in a short time. On the other hand, pcTBS needs less stimulation time than iTBS and 10Hz rTMS, which can greatly improve the efficiency of therapeutic instruments.

No significant changes were found for pain thresholds after one-session high-frequency rTMS stimulation, which supports the results reported in previously published studies (Antal and Paulus, 2010; Borckardt et al., 2011; Klírová et al., 2020). Klírová et al. found that pcTBS of the motor cortex can modulate cortical excitability but not pain perception. Antal and Paulus, and Borckardt et al. found that iTBS of the motor cortex did not induce a significant reduction in acute pain perception. However, most studies indicated that one-session high-frequency rTMS can decrease pain sensitivity (Ciampi et al., 2014; Moisset et al., 2015; De Martino et al., 2019; Liu et al., 2021). An explanation could be related to differences in the methodology used to assess pain thresholds. Most of the studies were conducted with pain induction tests using capsaicin, cold pain, heat pain, and pressure pain. Our study directly measures the pain tolerance threshold on sensory fibers of participants. Another explanation could be related to parameters of rTMS. A systematic review indicated the changes of MEP suppression in 30 Hz TBS were more persistent compared with 50 Hz TBS (Chung et al., 2016). In addition, one study found that three sessions of pcTBS to the left dorsolateral prefrontal cortex increased heat, cold, and pressure pain thresholds (De Martino et al., 2019). Therefore, frequency may be a key fact for rTMS to relieve pain.

### Acute Brain Activation Change After rTMS Stimulation

Although our behavioral data showed that one-session high-frequency rTMS can not increase the pain threshold of healthy subjects, fNIRS showed that high-frequency rTMS applied over left M1 can activate the DLPFC immediately after stimulation. After 1 h of stimulation, it can diffuse to the bilateral DLPFC and FPC. In addition, pcTBS can significantly activate DLPFC and FPC on A-fibers compared with iTBS, 10 Hz rTMS, and sham stimulation, which indicates that pcTBS has a potential analgesic effect. This result is in line with one study (Tupak et al., 2013). The authors indicated that iTBS applied to the left PFC can decrease prefrontal oxygenation. Other studies also used fNIRS to investigate acute neural adaptation after high-frequency rTMS on M1. However, decreased functional connectivity within PFC (Li et al., 2019) and reduction in HbO concentration from both motor and prefrontal cortices (Rihui et al., 2017) were observed during rTMS. The possible explanation is that these two studies applied rTMS over 1–2 cm lateral from the vertex rather than M1.

The analgesic effect of rTMS is still unclear. fMRI showed that rTMS can directly activate the thalamus through cortical-thalamic projection and inhibit the transmission of sensory information through the spinothalamic pathway, thus relieving pain (Cioni and Meglio, 2007). In addition, electrophysiological studies have shown that high-frequency rTMS can increase the excitability of the M1 area and cause cumulative plasticity changes of brain nerve tissue (Pridmore et al., 2005). Furthermore, studies indicated that chronic pain is accompanied by the decrease of blood perfusion in the thalamus and other parts, while low-frequency rTMS can reduce blood flow in the stimulated ipsilateral side and increase blood flow compensation in the contralateral brain (Tamura et al., 2004b). Our results indicate that single-session pcTBS placed on M1 can increase the excitability of DLPFC and FPC compared with iTBS, 10 Hz rTMS, sham stimulation. In addition, high-frequency rTMS can increase blood flow compensation in the prefrontal lobe. This result indicates that rTMS applied to the left M1 may play an analgesic effect by regulating DLPFC and FPC in the frontal lobe. More research is needed in the future to identify the interaction between M1 and the prefrontal cortex in pain research.

### Potential Clinical Application of pcTBS

One study examined the nociceptive threshold in the hind paws using the Neurometer in the bilateral carotid artery occlusion (BCAO) mouse model. The results found that the sensitivity of C and Aβ fibers (at stimulation of 5 and 2KHz, respectively) were significantly decreased in the 30 min BCAO group compared with those before BCAO, which were closely related to the development of the hyperalgesia component of central post-stroke pain (P) (Tamiya et al., 2013). Another study examined alterations of the current stimulation threshold of primary neurons using the Neurometer in mice receiving left middle cerebral artery occlusion (MCAO). The data showed that the sensitivity of Aδ and Aβ fibers (at 2 kHz and 250 Hz stimulation, respectively) was significantly decreased on day 3 after MCAO, which may contribute to the allodynia for CPSP (Takami et al., 2011). This study indicated that Aβ fiber damage may be the key to cause CPSP.

Our data show that pcTBS can modulate the sensitivity of Aβ fibers more effectively than 10 Hz rTMS and sham stimulation, which means that pcTBS may be used as a potential treatment for CPSP. In the future, multicenter, large-sample randomized controlled trials are needed to prove its effectiveness.

### Limitations

There are a few limitations in this study. Due to the limitation of research conditions, this study is the absence of a neuronavigation system that can target the left M1 according to the functional imaging examination. In addition, due to the limited number of fNIRS channels, we did not monitor the neuroplastic changes in the left M1. Therefore, we were unable to analyze the changes of brain functional connections between motor areas and the prefrontal cortex. In addition, sensory sensitivity may change at different ages. We only recruited subjects around 24 years old which may cause the results not stability across other age cohorts. Another limitation is that the sample of the study (*n* = 40) actually can be considered as a small sample, which can also limit the extrapolation of the results.

## Conclusion

In conclusion, our data demonstrate the advantages of pcTBS over 10 Hz rTMS and sham stimulation on Aβ fibers, opening new avenues of research concerning the clinical application of pcTBS for the treatment of CPSP. In addition, single-session pcTBS placed on left M1 can increase the excitability of DLPFC and FPC compared with iTBS, 10 Hz rTMS, and sham stimulation, indicating the interaction between M1 and prefrontal cortex may be a potential mechanism of analgesic effect of rTMS.

## Data Availability Statement

The raw data supporting the conclusions of this article will be made available by the authors, without undue reservation.

## Ethics Statement

The studies involving human participants were reviewed and approved by the Institutional Review Board of Huashan Hospital, Fudan University. The patients/participants provided their written informed consent to participate in this study. Written informed consent was obtained from the individual(s) for the publication of any potentially identifiable images or data included in this article.

## Author Contributions

CL, NZ, and ZW designed the experiment. CL, QH, LZ, and SX conducted the experiment. CL reduced and analyzed the data. CL and NZ wrote the manuscript. ZW revised the manuscript. All authors contributed to the article and approved the submitted version.

## Conflict of Interest

The authors declare that the research was conducted in the absence of any commercial or financial relationships that could be construed as a potential conflict of interest.

## Publisher’s Note

All claims expressed in this article are solely those of the authors and do not necessarily represent those of their affiliated organizations, or those of the publisher, the editors and the reviewers. Any product that may be evaluated in this article, or claim that may be made by its manufacturer, is not guaranteed or endorsed by the publisher.
